# Editorial: Clinical application and impact of blood-flow-restriction training

**DOI:** 10.3389/fphys.2023.1155080

**Published:** 2023-02-20

**Authors:** Michael Behringer, Alexander Franz, Luke Hughes

**Affiliations:** ^1^ Department of Sports Sciences, Goethe University Frankfurt, Frankfurt am Main, Germany; ^2^ Department of Orthopedics and Trauma Surgery, University Hospital Bonn, Bonn, Germany; ^3^ Department of Adult Reconstruction, ATOS Orthoparc Clinic Cologne, Cologne, Germany; ^4^ Department of Sport Exercise and Rehabilitation, Northumbria University, Newcastle upon Tyne, United Kingdom

**Keywords:** blood flow restriction (BFR), tendinopathic tendons, adverse (side) effects, arthritis, total knee anthroplasty, heart disase, sarcopenia, prehabilitaion

For a long time, it was firmly believed that a high load in strength training was necessary to achieve muscle mass and strength gains. However, blood flow restriction training has fundamentally challenged this assumption. Numerous studies over the past decades have shown that these biopositive effects can be achieved even with low loads when blood flow to the working muscles is restricted and venous return from the working extremity to the heart is interrupted (Labata-Lezaun et al., 2022; Perera et al., 2022).

This observation is not only a groundbreaking discovery from a muscle physiology perspective but also offers numerous possibilities in the clinical setting from a practical perspective. In this setting, physicians and therapists were often faced with the dilemma that recommendations for preserving or improving musculature through classical strength training with high loads were opposed by the reduced load-bearing capacity of the patients’ musculoskeletal system. While BFR training in healthy individuals has been studied extensively in the sports science context, the evidence in the clinical setting is comparatively limited (Hughes et al., 2017). For this reason, the initiation of the Research Topic was an important step in advancing knowledge in this field. The aims and objectives were to expand current knowledge about the feasibility and effects of BFR applications in the clinical setting. This was not only to broaden the range of BFR applications, but also to shed light on possible negative effects of BFR training in patient populations in addition to the positive ones, thus providing a scientific basis for future work.

A total of 35 experts participated in this Research Topic and presented the results from their current investigations. A total of nine studies were accepted to be published in the Research Topic.

Burton and McCormack focus their scoping review on the effects of BFR training on tendon injuries and healthy tendons. They conclude that the limited results to date on this topic are encouraging and that further research in this area is likely merited. Høgsholt et al. also dealt with BFR training and tendons in their work. However, in their feasibility study, the researchers highlighted the effect of BFR training in combination with patient education on gluteal tendinopathy and were able to show that BFR training brought about an improvement in strength and pain. However, the authors also pointed out that they had one participant who dropped out of the study due to an adverse event in the form of pain below the applied cuff. Even though the affected person was able to resume her daily exercise sessions without the LL-BFR cuff after only 2 weeks, it makes it clear that BFR training can certainly be associated with risks. All the more important are the remarks of Nascimento et al. They point out that a medical history should be taken before starting BFR training to identify such pathologies or comorbidities that could be associated with adverse side effects. For this purpose, they present a model in their publication that could be used in practice.

The studies in this Research Topic have demonstrated that BFR training can be used successfully in a variety of clinical settings. Jørgensen and Mechlenburg report in their case-study that BFR training was able to improve functional performance and reduce swelling in a 17-year old male, suffering from reactive arthritis after a 12-week low load BFR-Training even with low amounts of supervision. In a study by our own research group, we were able to show that BFR training is also a very effective tool in the prehabilitation phase (Franz et al.) Here, too, the patients’ load bearing capacity is often reduced and traditional strength training with high loads is unsuitable. After 6 weeks of prehabilitation with BFR prior to total knee arthroplasty implantation, patients’ muscle mass, strength, and quality of life (QoL) improved. Cahalin et al. performed a comprehensive literature review on the potential benefits of BFR-Training in heart disease and heart failure. On the basis of current data, BFR training for patients with different cardiac diseases and heart failure not only appears to be safe but also seems to improve numerous parameters, such as left ventricular dysfunction, inflammatory markers, dyspnea, fatigue, and peripheral blood flow. BFR training could also be beneficial for individuals who suffer from sarcopenia preventing them from performing traditional moderate to high load resistance training, or for whom such training would even be associated with an increased risk of injury. Cahalin et al. used a systematic review and meta-analysis to determine whether data have already been published on this topic. Although the authors could not find any studies that explicitly dealt with BFR training in sarcopenia patients, they did find numerous functional improvements in older people, making BFR training interesting for sarcopenia patients. This study highlights the importance of employing inclusion criteria specific to sarcopenia when this is the target population.

As Cuffe et al. showed in a survey-based study of current trends in BFR training, there is a great deal of diversity in the use of BFR training. Not only did users come from different professions, but the equipment used and the training design varied significantly. However, the appropriate devices are indispensable for accurate BFR-application. Citherlet et al. compared two different cuffs and concluded that both failed to accurately modulate blood flow.

We are convinced that the results of this Research Topic will help practitioners and researchers to improve the application of BFR-Training in the clinical setting and to identify relevant research gaps. It is evident, not least from the Research Topic of articles in this Research Topic, that the application of BFR in the clinical setting is an exciting new area of research. However, as a result of its novelty, many questions remain unanswered. Future studies need to shed more light on potential negative side effects, especially in people with pre-existing conditions. Regarding knowledge of the effects of BFR training on the cardiovascular system and passive musculoskeletal system in particular, we are still in our infancy. Even though two of the studies in this Research Topic dealt with the effects on tendon tissue, it must be stated that we still know too little about the effects of BFR training on the passive musculoskeletal system. Also, the possible positive as well as negative effects of BFR training on muscle diseases have not been researched much yet. Furthermore, it is critical that future research implements BFR according to our current understanding of optimal parameters of application (Patterson et al., 2019). Thus, BFR training in the clinical setting remains an interesting Frontier of research.

## References

[B1] Hughes L. Paton B. Rosenblatt B. Gissane C. Patterson S. D. (2017). Blood flow restriction training in clinical musculoskeletal rehabilitation: A systematic review and meta-analysis. Br. J. Sports Med. 51, 1003–1011. 10.1136/bjsports-2016-097071 28259850

[B2] Labata-Lezaun N. Llurda-Almuzara L. González-Rueda V. López-de-Celis C. Cedeño-Bermúdez S. Bañuelos-Pago J. (2022). Effectiveness of blood flow restriction training on muscle strength and physical performance in older adults: A systematic review and meta-analysis. Archives Phys. Med. Rehabilitation 103, 1848–1857. 10.1016/j.apmr.2021.12.015 35026149

[B3] Patterson S. D. Hughes L. Warmington S. Burr J. Scott B. R. Owens J. (2019). Corrigendum: Blood flow restriction exercise: Considerations of methodology, application, and safety. Front. Physiology 10, 1332. 10.3389/fphys.2019.01332 PMC6818375 31695626

[B4] Perera E. Zhu X. M. Horner N. S. Bedi A. Ayeni O. R. Khan M. (2022). Effects of blood flow restriction therapy for muscular strength, hypertrophy, and endurance in healthy and special populations: A systematic review and meta-analysis. Clin. J. Sport Med. 32, 531–545. 10.1097/JSM.0000000000000991 36083329

